# What approaches to social prescribing work, for whom, and in what circumstances? A protocol for a realist review

**DOI:** 10.1186/s13643-016-0269-6

**Published:** 2016-06-03

**Authors:** Kerryn Husk, Kelly Blockley, Rebecca Lovell, Alison Bethel, Dan Bloomfield, Sara Warber, Mark Pearson, Iain Lang, Richard Byng, Ruth Garside

**Affiliations:** NIHR CLAHRC South West Peninsula (PenCLAHRC), Plymouth University Peninsula Schools of Medicine and Dentistry, Plymouth University, Plymouth, England; European Centre for Environment and Human Health, University of Exeter Medical School, University of Exeter, Exeter, England; NIHR CLAHRC South West Peninsula (PenCLAHRC), University of Exeter Medical School, University of Exeter, Exeter, England; College of Life and Environmental Sciences, University of Exeter, Exeter, England; University of Michigan Medical School, University of Michigan, Ann Arbor, MI USA

**Keywords:** Social prescribing, Primary care, Mental health, Community referral, Realist review

## Abstract

**Background:**

The use of non-drug, non-health-service interventions has been proposed as a cost-effective alternative to help those with long-term conditions manage their illness and improve their health and well-being. Interventions typically involve accessing activities run by the third sector or community agencies and may also be described as non-medical referral, community referral or social prescribing. To be effective, patients need to be “transferred” from the primary care setting into the community and to maintain their participation in activities. However, it is not currently known how and why these approaches enable which people under what circumstances to reach community services that may benefit their health and well-being.

**Methods:**

Database searches and extensive searching of grey sources will be carried out in an attempt to find evidence associated with referral and retention in social prescribing. After initial scoping searches, two main phases of searching will be conducted: (a) will focus on the identification of programme theories to illustrate how approaches to social prescribing work for different people and in different contexts and (b) will consist of targeted searches to locate evidence to refine these candidate theories into configurations of the contexts in which populations and the main mechanisms outcomes are achieved. Inclusion criteria will initially be broad in order to develop a clear picture of the ways in which social prescriptions might operate but may iteratively become more focused in response to initially identified evidence, for example, in terms of the population group.

An expert advisory group consisting of professionals working in a range of organisations involved in social prescribing will be convened to check the approaches in the review and provide real-life experience of social prescribing. Findings from the review will be disseminated to commissioners, published in a peer-reviewed journal and used to help refine an intervention model for an outdoor nature-based group intervention.

**Discussion:**

This realist review will explore why mechanisms of social prescribing work, for what groups of people and their impact on enrolment, attendance and adherence to programmes. The use of realist approaches to detail the social prescribing process is novel and will offer insights into effective transfer of patients.

**Systematic review registration:**

PROSPERO CRD42016039491

## Background

The use of non-drug, non-health-service interventions has been proposed as a cost-effective alternative to help those with long-term conditions manage their condition and improve their health and well-being [1], contributing to the global “Triple Aim” by applying integrated approaches to simultaneously improve care, improve population health, and reduce costs per capita [2]. Typically, this involves accessing activities run by the third sector or community agencies and is described as non-medical referral, community referral or social prescribing^1^ [3]. As of yet, there is no agreed definition of social prescribing [4]. Patients may self-refer or be referred through a healthcare or other professionals. A wide range of social prescription activities have been developed, including art therapy, walking groups, reading groups, nature-based activities and volunteering [5]. These are multi-component, complex interventions and are similar to more established exercise referral schemes [6–8]. Mild to moderate depression is one condition that may benefit from social prescribing, particularly as the third most common reason for general practice consultations in the UK is depression [9]. Approximately 1 in 20 people experience an episode of depression each year, with one quarter of women and 1 in 10 men requiring treatment for it at some point over their lifetime [9]. The causes of depression can be wide-ranging, with symptoms including social isolation, a decrease in activity, sleep disturbances, incapacity to work, poor concentration and poor eating habits [10].

Social prescribing interventions potentially enable healthcare providers to respond to a more holistic range of patient needs, reduce frequency of attendance to GPs, reduce social isolation and aid return to work. It is also argued that social prescription models extend the traditional boundaries of primary care and facilitate opportunities for contact with dedicated professionals with increased time allocation and more specific expertise [11–14]. The current health policy and guidance, such as the English NHS’s Five Year Forward View [15] and the Social Value Act [16], are supportive of the types of mechanisms and approaches inherent to social prescribing models.

For social prescription interventions to be successful, patients need to be successfully “transferred” from the primary care setting to the relevant resource and to maintain participation for an appropriate period of time. By primary care, we include community mental health and general practice services. The routes through which people are referred are likely to influence the uptake of these services and therefore their success. For instance, without an appropriate, supportive structure for this transition, the suggestion (i.e. referral) to pursue an activity is unlikely to be taken up by the patient [4, 17]. Workshops hosted by Bromley PCT established the following six models of transfer [4, 5]:

1. Information service—this is an information-only service, with display boards and directory access. There is no face-to-face contact.
2. Information and telephone line—leaflets and notice boards advertise the service, and patients self-initiate a telephone discussion with a worker. Again, there is no face-to-face contact.
3. Primary care referral—primary healthcare workers assess patients and refer them to a social prescribing service. This is based upon an appointment leading onto non-clinical issues and is opportunistic.
4. Practice-based generic referral worker—groups are held in general practice, and patients can be referred by health workers or self-refer. This may mean that there is a process of triage and signposting. The surgery can act as a “one-stop shop”.
5. Practice-based specialist referral worker—as above the specialist, worker works from primary care practices but may offer specific services such as Citizens Advice. Direct advice may then be offered as well as referral or signposting onwards.
6. Non-primary care based—referrals from practice-based staff are sent to a referral centre (Bromley User Group Outreach Centre). This could be an outreach service, set in the community, offering a one-to-one service. Other organisations also use this service and facilities.

Others have suggested that effective delivery would need to consider the following: “link” workers, resource directories, developing of primary care organisational links, increased referrals to organisations, cultural differences between sectors and diversity of referral routes [4, 11, 12, 14].

A recent rapid review found little good quality evidence about social prescribing, largely due to a paucity of controlled study designs [18]. However, the review conflated the health and well-being impact of the activities themselves and the prescribing route through which they are reached. Success is likely to relate to the interaction between the ways in which people are referred and supported, the context in which referral and the activities are offered and the needs of the people referred as well as the health and well-being impacts of the activities to which they are referred. There is a lack of systematic consideration of the approaches used to enable people to reach community services, to support continuing participation, or whether some approaches are more effective or cost-effective than others [4].

We plan to undertake a realist review to explain why different methods of referral and retention in social prescribing activities do (or do not) work in certain circumstances for certain populations as well as the uncertainties relating to methods of referral and retention [19]. A realist review, or realist synthesis, seeks to answer specified research questions by applying a realist philosophy of science to the synthesis of relevant evidence. A realist philosophy is based on the principle that intended and unintended outcomes of interest (O) are generated through the interaction between context (C) and mechanism(s) (M). Consequently, what works well for one group of people may not work at all in different circumstances or for a different group of people [20]. The use of this method will provide understanding of causal mechanisms and, specifically, what impacts the social context may have on shaping the decisions made and therefore of the outcomes of the social prescribing process. The method has previously been used, by members of the review team, to understand the implications of contexts and mechanisms on the outcomes of other forms of health promotion interventions or delivery strategies [21–23].

### Review objectives

The primary objective of this review is to explain why different methods of referral and encouraging adherence to social prescribing activities do (or do not) work in certain circumstances for certain populations. The review will have two phases in approaching the objectives listed below: first, programme theories in the literature will be identified, and second, targeted searches will be used to seek suitable evidence to refine these theories (see the “Methods” section).

1. What are the main factors or mechanisms^2^ that are thought (both scientifically and experientially) to explain the success or failure of social prescribing? (We are defining success as enrolling in an activity, turning up for the first session and continuing with the activity, as per outcomes 1–3 below. The activity will be deemed as failing if one or more of these three conditions are not met.)
2. Are there methods of referral that are particularly useful and appropriate for different groups of people (we are particularly interested in those with mild to moderate depression)?
3. Which approaches to referral are likely to be appropriate, or inappropriate, within NHS primary care settings?
4. What is the best practice for people with mild to moderate depression to receive nature-based interventions? (By this, we mean any intervention which occurs in the natural environment where, for example, greater benefits have been shown to accrue compared to similar activities indoors [24, 25]).

## Methods

As we will explore the complex nature of social prescriptions, the methods of the realist review methodology are most suitable [19]. Realist approaches try to understand, by seeking to explain causation and how causal mechanisms are shaped and constrained by the social context, “What works, how, why, for whom, to what extent and in what circumstances, in what respect and over what duration?” Realist reviews usefully draw on evidence from a wider range of sources than traditional systematic reviews.

### Search strategy

Initial scoping searches will be conducted to develop familiarity with the various models of social prescribing relevant to NHS primary care settings. Subsequently, iterative and progressively more focussed searches will then be used through two main phases.

First (a) is the identification of programme theories, which will be used to illustrate how approaches to social prescribing are thought to work for different groups of people in different contexts. Since remaining involved in the activity is important, as well as being referred to it, we will consider contextual factors such as the suitability and acceptability of the types of activity to which people are referred. We will use this range of candidate theories to develop a series of “if, then” statements around mechanisms which we will refine through discussion.

Second (b), following the development of key programme theories, targeted searches will be undertaken to seek evidence suitable to refine these candidate theories.

Search terms for (a) will focus on types of prescribing models and also the setting (e.g. GP, community provider). Search terms for (b) will depend on the results from the first phase and will be discussed both within the review team and the broader expert advisory group for sense-checking and completeness.

Electronic databases to be searched in both phases will be as follows:

- MEDLINE
- Embase
- CINAHL
- GreenFILE
- PsycInfo

The purpose of the searches is not to be exhaustive, but rather to purposively identify a range of models which, coupled with the fact that we anticipate the literature for this topic to be diverse and dispersed, means we will use extensive searching of grey sources. The precise approach will develop alongside the project, however we anticipate using top-level website searches (potential models might be provided by The Conservation Volunteers, National Trust, Natural England and others), selected organisation contacts through the expert advisory group, and searches of, for example, OpenGrey.

We will also undertake forward and backward citations chasing on included studies, hand-searching of key journals, and study author contact at both stages where appropriate.

### Study inclusion criteria

In order to develop a clear picture of the range of ways in which social prescriptions might operate, we will include evidence, in the initial phase (a—as above), which is of relevance and contributes to theory building and testing through provisions of descriptions of how potential participants are identified, nature of referral model, people involved in the referral model, context for referral, nature and type of support offered following referral and so on. These sources of evidence are likely to include but not limited to editorials, opinion pieces, communications, primary studies, process evaluations and systematic reviews. Screening will likely be iterative, with disagreements providing articulation of study richness and relevance, on which prioritisation of studies will be based should it be required.

#### Population inclusion criteria

In the second phase (b), we will include any studies which include adults (>18) in a primary care setting. By primary care, we incorporate community mental health services, home care, community pharmacies and services in general practices. Referral can be through any health professional or dedicated link worker based in general practice. Initially, we will not restrict evidence to those with mild to moderate depression in order to obtain information on all potentially relevant social prescribing models, but we may iteratively focus down the population groups in response to initially identified evidence. For example, we may focus on a group exhibiting key personality traits associated with depression (anxiety, social isolation) where there is a particularly rich seam of evidence.

#### Intervention inclusion criteria

Social prescribing programmes which have sought to transfer patients between primary care and organisations delivering community-based activities. As above, we will initially cast a wide net to ensure that we can learn from all available prescribing models, including exercise referral, arts based practice and nature-based referrals.

#### Comparator

As a range of evidence and study designs will be included, comparator criteria will only be applied to comparative designs, for example, in randomised controlled trials which compare social prescribing to a non-social prescribing activity. Relevant comparator activities will be those delivered within or through primary care (i.e. patients are not referred out to a community-based activity), and examples will be specific to conditions but may include prescriptions for drugs, cognitive behavioural therapy or physiotherapy. Only the processes of prescription and acceptance, attendance or adherence will be compared, rather than any effect the treatment or intervention has on the patient’s health.

#### Types of study

As stated, in the first phase (a), we will include evidence that provides descriptions of social prescribing processes; we anticipate that important details will be provided in non-empirical studies.

We will include, in the second phase (b), quantitative studies including randomised controlled trials (RCTs), controlled before-and-after studies, interrupted time series, cohort studies and case-control studies. However, any study providing a detailed account of a programme will be considered.

We will include qualitative studies from any discipline or theoretical tradition that uses recognised methods of data collection and analysis. We will also include qualitative evidence linked to quantitative intervention studies (“sibling studies”).

Quantitative or qualitative process evaluations will be included.

Where insufficient controlled evidence is located, we will include uncontrolled studies (including, for example, uncontrolled before-and-after studies).

#### Outcomes

We anticipate that the outcomes will be diverse and context specific, and so we are unable to produce an exhaustive list at the outset. The outcomes will relate to the process of social prescribing and are likely to encompass the following:

1. Primary care professionals’ awareness and prescribing of a social prescription activity and patients’ acceptance of the prescription (enrolment)
2. Patients’ initial participation in the activity (engagement)
3. Patient’s ongoing engagement and/or completion of the prescription (adherence)

“Contexts” will be central to the interpretation of the outcomes, and their impact to the firing of particular mechanisms will be of interest to the review (physician-led “prescription” versus patient-led “candidacy of treatment”, for example).

The *effectiveness* of the social prescriptions activities on health outcomes will not be considered in this review.

We will explore relevant outcome measures with our expert advisory group and with the full-review team.

### Selection of studies

Titles and abstracts (where available) will be screened by one reviewer, and where these appear to fit the inclusion criteria, the full text will be screened by two reviewers. Disagreements will be resolved through discussion and, where required, a third reviewer.

### Quality assessment

We will assess the rigour (i.e. whether the method used to generate that particular piece of data is credible and trustworthy) of relevant evidence in the following ways: in the first phase (a) and in keeping with other realist reviews, we will use a hybrid classification tool in the first instance [26], which classifies studies as either conceptually rich, thick, or thin.

In the second phase (b), we will use standard quality assessment tools suitable to judge the plausibility and coherence of the method used to generate data in the included studies. For randomised studies, we will use the Cochrane Collaboration’s tool for assessing risk of bias [27], and for non-randomised studies and qualitative studies, we will use the Wallace criteria [28] for assessing both reporting and methodological standards.

Appraisal of studies at both stages will be undertaken by two reviewers independently, with any disagreement being resolved through discussion and where necessary a third reviewer.

### Data extraction

The exported files from the searching will be uploaded and de-duplicated in Endnote X7 [29]. Searches will be recorded using the Preferred Reporting Items for Systematic Reviews and Meta-Analyses (PRISMA) guidelines [30].

In the first phase (a), data will not be formally “extracted” but rather, in keeping with a realist approach [26], the review team will engage with the evidence through note-taking, annotation and discussion. Specifically, we will examine the processes of social prescribing for health and well-being and factors contributing to success. These processes will help develop programme theory. The data extraction process will be continually refined based on these discussions between the review team and the themes emerging from the included studies. We will organise and analyse data at this stage, if appropriate, using NVivo 10 software [31].

The second phase (b) will build on the first phases to ensure relevant data is captured and, in method, will constitute more traditional data extraction. Data will be extracted into bespoke forms developed for the review. Data will be extracted by one reviewer and checked by another. We will extract data which helps clarify and explain the mechanisms and refine programme theory. Extracted data will likely relate to full details of the nature of the programme (prescription process, information given, activity, time frame and frequency of engagement) and any theory informing it, setting/provider, and outcomes.

### Expert advisory group

An expert advisory group will be convened to check our approaches and to develop “everyday” theories about how social prescribing is thought to work, or not work, in different contexts for different groups of people. This advisory group will meet at least once in person over the course of the project. It will comprise general practice staff involved in social prescribing, community/third sector groups providing activities that are socially prescribed and specialist academics in the field.

### Synthesis

We aim to build an understanding of the process of social prescribing by identifying how specific outcomes are generated by relevant mechanisms which are triggered in particular contexts. We will seek recurring patterns across the data.

We will use the same approach to synthesis for both phases (a) and (b), following the realist methodology [21, 32]:

- Juxtaposition of sources of evidence—for example, where evidence about implementation in one source enables insights into evidence about outcomes in another source.
- Reconciling of sources of evidence—where results differ in apparently similar circumstances; further investigation is appropriate in order to find explanations for why these different results occurred.
- Adjudication of sources of evidence—on the basis of methodological strengths or weaknesses.
- Consolidation of sources of evidence—where evidence about mechanisms and outcomes is complementary and enables a multi-faceted explanation to be built.
- Situating sources of evidence—where outcomes differ in particular contexts; an explanation can be constructed of how and why these outcomes occur differently.

We will organise studies according to mechanism and contextual factors such as type of activities, type of participants, modes of referral and support. We will further explore mechanisms and complex outcomes through tabulation of data and, where appropriate, through the derivation and iterative development of a conceptual framework/logic model [33]. We will ensure that the limitations of the methods used to generate included evidence are identified and taken into consideration throughout the synthesis. Transparency will be ensured by documenting the synthesis approach and process.

## Dissemination

We will submit the results of the review to a high-impact, peer-reviewed journal. Any write-up will follow the Realist And Meta-narrative Evidence Syntheses: Evolving Standards (RAMESES) publication guidelines [20].

We will also produce a briefing document for commissioners outlining our findings. This will be a two-page summary of the purpose, aims, findings, and implications of the review that are relevant and user-friendly for the commissioners’ respective organisations.

In addition, the findings will help to refine an intervention model about nature-based interventions (NatureScript) for those with mild to moderate depression. This approach is being offered by a number of GPs in the South West under the Dose of Nature scheme, as well as through other third sector providers, such as the Conservation Trust’s Green Gym.

## Abbreviations

GP, general practitioner; NHS, National Health Service; NIHR, National Institute for Health Research; PCT, Primary Care Trust; PenCLAHRC, Peninsula Collaboration for Leadership in Applied Health Research and Care; PRISMA, Preferred Reporting Items for Systematic Reviews and Meta-Analyses; RAMESES, Realist And Meta-narrative Evidence Syntheses: Evolving Standards; RCT, randomised controlled trial.

## Footnotes

1 “Social prescribing” might refer to “non-healthcare” interventions addressing a range of social and health problems and may be social in nature (e.g. groups) but could also be activities aiming to increase connectedness with place and/or activity as well as people.

2 Mechanisms are conceptualised here as the way in which a programme’s resources or opportunities interact with the reasoning of individuals and lead to changes in behaviour. These relate to or are synonymous with “intervention functions” [34].
